# Supplementary material for: Structure and elevator mechanism of the mammalian sodium/proton exchanger NHE9
Source: EMBO J. 2020 Oct 29;39(24):e105908. doi: 10.15252/embj.2020105908 (PMC7737618; doi:10.15252/embj.2020105908)
Supplement: Supplementary file 1 — Appendix [file EMBJ-39-e105908-s001.pdf]

Appendix for

Structure and Elevator Mechanism of the Mammalian Sodium/Proton Exchanger NHE9

Iven Winkelmann[1*], Rei Matsuoka[1*], Pascal F. Meier[1*], Denis Shutin[2], Chenou Zhang[3], Laura Orellana[1], Ricky Sexton[3], Michael Landreh[4], Carol V. Robinson[2], Oliver Beckstein[3], David Drew[1#]

Table of Contents:

- Appendix Table S1-S2
- Appendix Figures S1-S12 (incl. figure legends)

Appendix Table S1

ID	atoms	run length (ns)	protomer A protonation state	protomer A binding probability	protomer B protonation state	protomer B binding probability
m1-10-f-1	160475	698.01	S1	0%	S0	0%
m1-10-f-2	160475	520.62	S1	0%	S0	0%
m1-10-f-3	160475	109.38	S1	0%	S0	0%
m1-10-p0-1	160475	348.76	S1	0%	S0	95%
m1-10-p0-2	160475	201.74	S1	0%	S0	100%
m1-10-p0-3	160475	126.15	S1	0%	S0	9%
m1-10-p-1	160475	299.38	S1	0%	S0	8%
m1-10-p-2	160475	304.45	S1	0%	S0	33%
m1-10-p-3	160475	252.97	S1	0%	S0	51%
m1-32-f-1	136784	186.62	S3	0%	S2	0%
m1-32-f-2	136784	95.3	S3	0%	S2	0%
m1-32-f-3	136784	293.63	S3	0%	S2	0%
m1-32-p-1	136784	198.68	S3	0%	S2	1%
m1-32-p-2	136784	204.53	S3	0%	S2	0%
m2-00-f-1	130949	577.25	S0	0%	S0	0%
m2-00-f-2	130949	670	S0	0%	S0	0%
m2-00-f-3	130949	610.47	S0	85%	S0	0%
m2-00-p-1	130949	470.86	S0	11%	S0	100%
m2-00-p-2	130949	512.35	S0	53%	S0	100%

Summary of all-atom MD simulations. **ID**: name of the simulation [includes m1/m2: model M1/M2 as described in the text; protonation states of protomers A and B (S0–S3, see below); if binding site was modelled with a Na^+ ion included ("p" for pre-bound) or if it was left empty and ions could freely diffuse ("f") or "p0" if protomer A was left empty and protomer B was pre-bound; repeat number of simulation (1–3)]. **Atoms:** number of atoms in the simulation. **Run length**: simulated time in ns. For **protomer A** and **protomer B** of the dimer, different protonation states of Asp244 and Asp215 were simulated: **S0** (both deprotonated), **S1** (Asp244 protonated, Asp215 deprotonated), **S2** (Asp244 deprotonated, Asp215 protonated), **S3** (both protonated). **Binding probability**: fraction of total run time during which a Na^+ ion was bound to Asp244 as described in the text.

Appendix Table S2

Transport domain – dimer domain interaction	
**Protein**	**Buried Surface Area (Å^2)**
NHE9 CTD	962
NapA *inward*	1193
NhaA inward	1215
*Pa*NhaP pH4 *inward*	1341
*Mj*NhaP pH4 *inward*	1475
*Pa*NhaP pH8 *inward*	1528

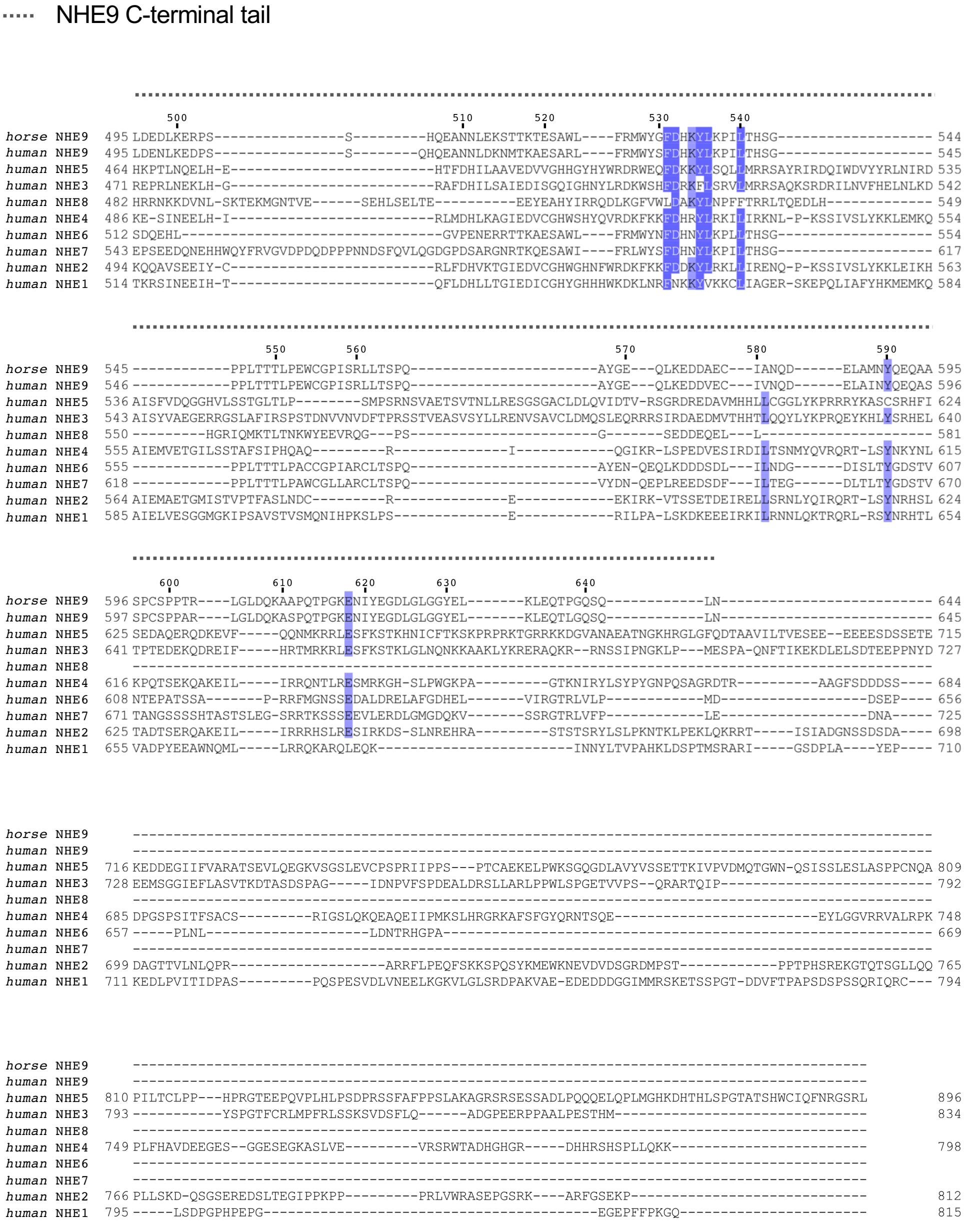

Appendix Figure S1. Multiple sequence alignment of *horse* NHE9 and *human* NHE1-9 isoforms. Alignment shown is for the C-terminal regulatory domain, which has been continued from the transporter domain alignment shown in Fig. EV1. Residues with over 70% sequence identity are indicated by purple background.

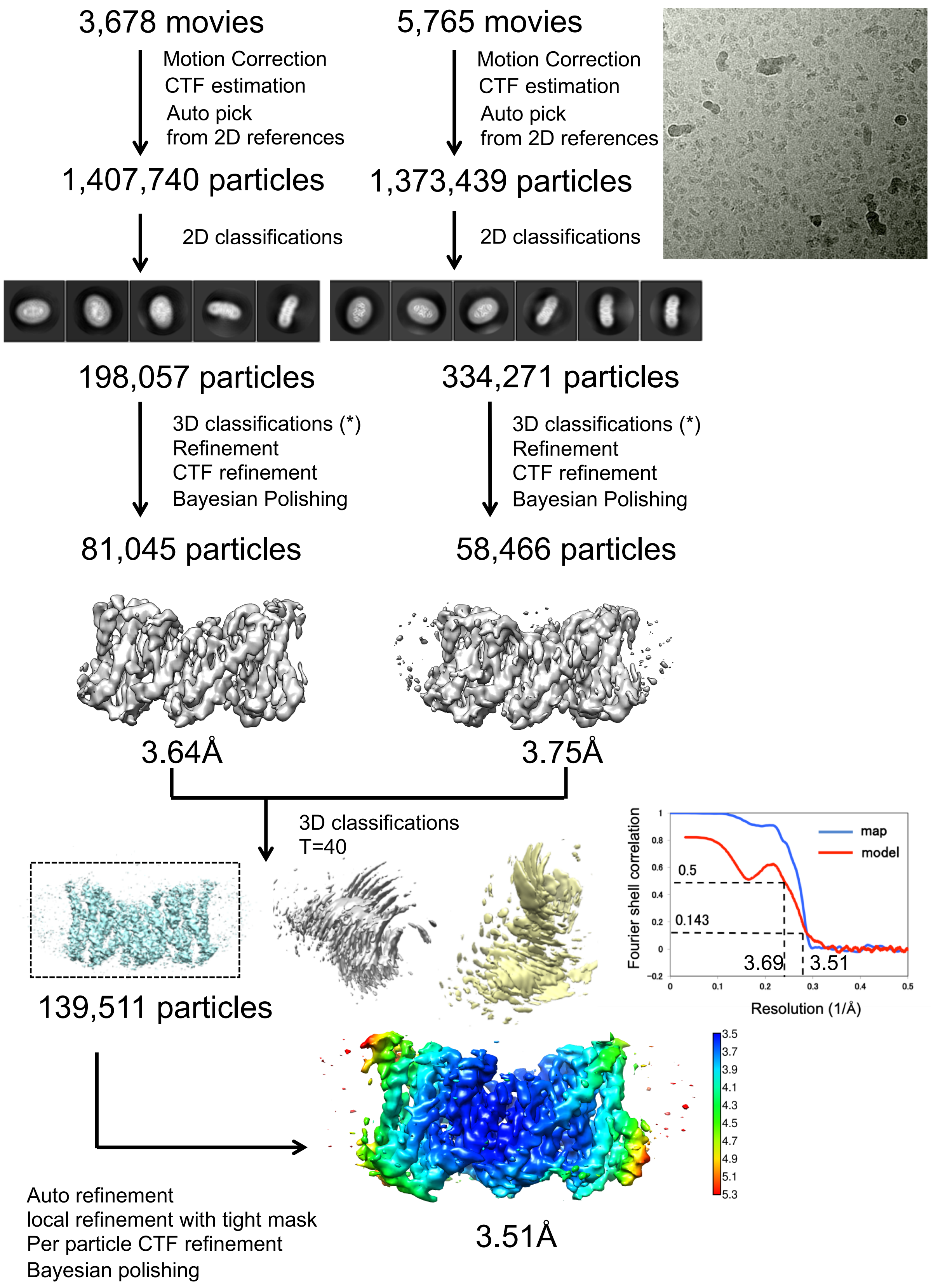
3,678 movies
Motion Correction
CTF estimation
Auto pick
from 2D references
1,407,740 particles
2D classifications
198,057 particles
3D classifications (*)
Refinement
CTF refinement
Bayesian Polishing
81,045 particles
3.64Å
5,765 movies
Motion Correction
CTF estimation
Auto pick
from 2D references
1,373,439 particles
2D classifications
334,271 particles
3D classifications (*)
Refinement
CTF refinement
Bayesian Polishing
58,466 particles
3.75Å
3D classifications
T=40
139,511 particles
Fourier shell correlation
Resolution (1/Å)
map
model
0.5
0.143
3.69
3.51
Auto refinement
local refinement with tight mask
Per particle CTF refinement
Bayesian polishing
3.51Å

Appendix Figure S2. The data-processing workflow of horse NHE9*. An initial dataset contained 3,678 movies that were corrected by MotionCor2 and CTFFind. After reference-based auto-picking, 1,407,740 particles were picked. Several rounds of 2D classification were performed, yielding 198,057 particles, which were subjected to 3D classification. One of three 3D classes was selected and this class contained 81,045 particles. A second dataset was collected and the same procedure carried out and the resulting best 3D class contained 58,466 particles. A total of 139,511 particles were merged and the 3D classification repeated to remove noise particles, *i.e*, without this additional round of 3D classification, the reconstructed map was suffering from white noise. After several rounds of refinement with global and local search using ctf refine and polishing, a final resolution of 3.51Å was achieved at the gold standard FSC (0.143), with a local resolution range of 3.5 to 4.9Å.

A

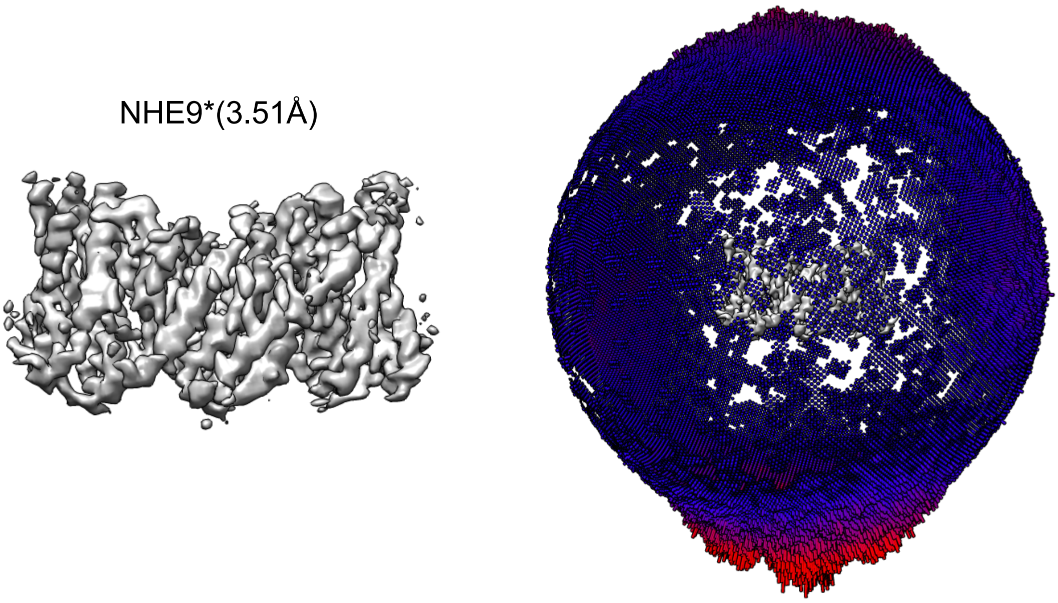

B

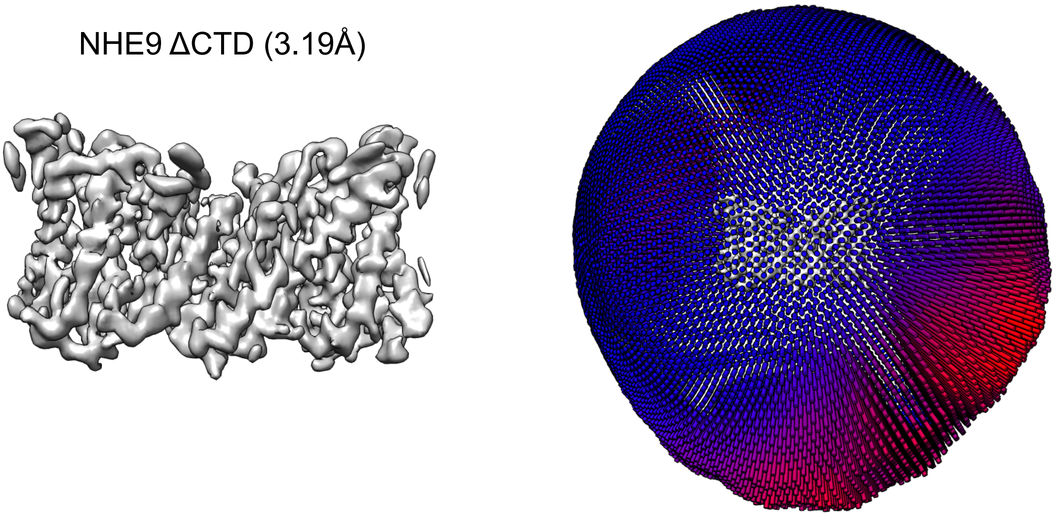

Appendix Figure S3. Distribution of NHE9* and NHE9 ΔCTD particle orientation. (A) The cryo-EM map of NHE9* (left) and the distribution for orientation sampling after refinement that was sampled by 0.46785 degree (right). **(B)** As in (A) for NHE9 ΔCTD that was sampled by 0.9375.

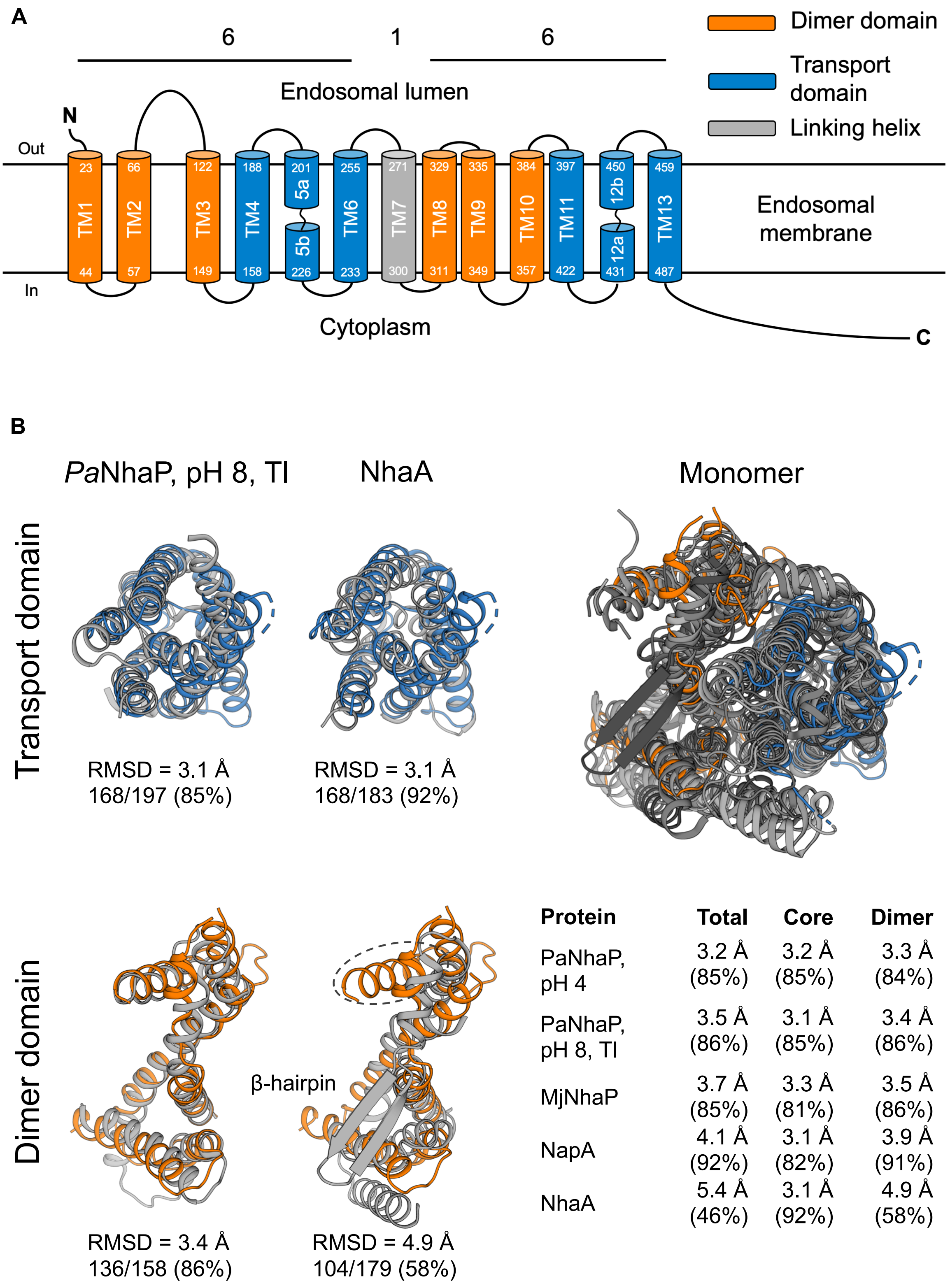

Protein	Total	Core	Dimer
PaNhaP, pH 4	3.2 Å (85%)	3.2 Å (85%)	3.3 Å (84%)
PaNhaP, pH 8, TI	3.5 Å (86%)	3.1 Å (85%)	3.4 Å (86%)
MjNhaP	3.7 Å (85%)	3.3 Å (81%)	3.5 Å (86%)
NapA	4.1 Å (92%)	3.1 Å (82%)	3.9 Å (91%)
NhaA	5.4 Å (46%)	3.1 Å (92%)	4.9 Å (58%)

Appendix Figure S4. NHE9 topology, structure and comparison to bacterial homologues. (A) NHE9 consists of 13 TMs that are arranged into two 6 TM structural-inverted repeats, which intertwine to form the dimerization domain (orange) and 6-TM core transport domains (blue) respectively, connected by the TM7 linking helix (grey). The cytosolic C-terminal regulatory domain (CTD) is 157 residue long. **(B)** *left*: cartoon representation showing the structural superimposition of the respective domains of the NHE9 monomer, dimer domain only (orange), and transport domain only (blue), against bacterial homologues structures (grey). The dashed circle highlights TM1 in NHE9, which is missing from NhaA. *right*: shows the superimposition of the entire monomers of the respective structures, with the associated r.m.s.d values calculated by CEalign in PyMol and number of Cα atoms aligned. Note only 58% of the NhaA dimer domain could be aligned to the NHE9 dimerization domain as it contains one less TM than NHE9 (orange and dotted circle) and beta hairpins (grey).

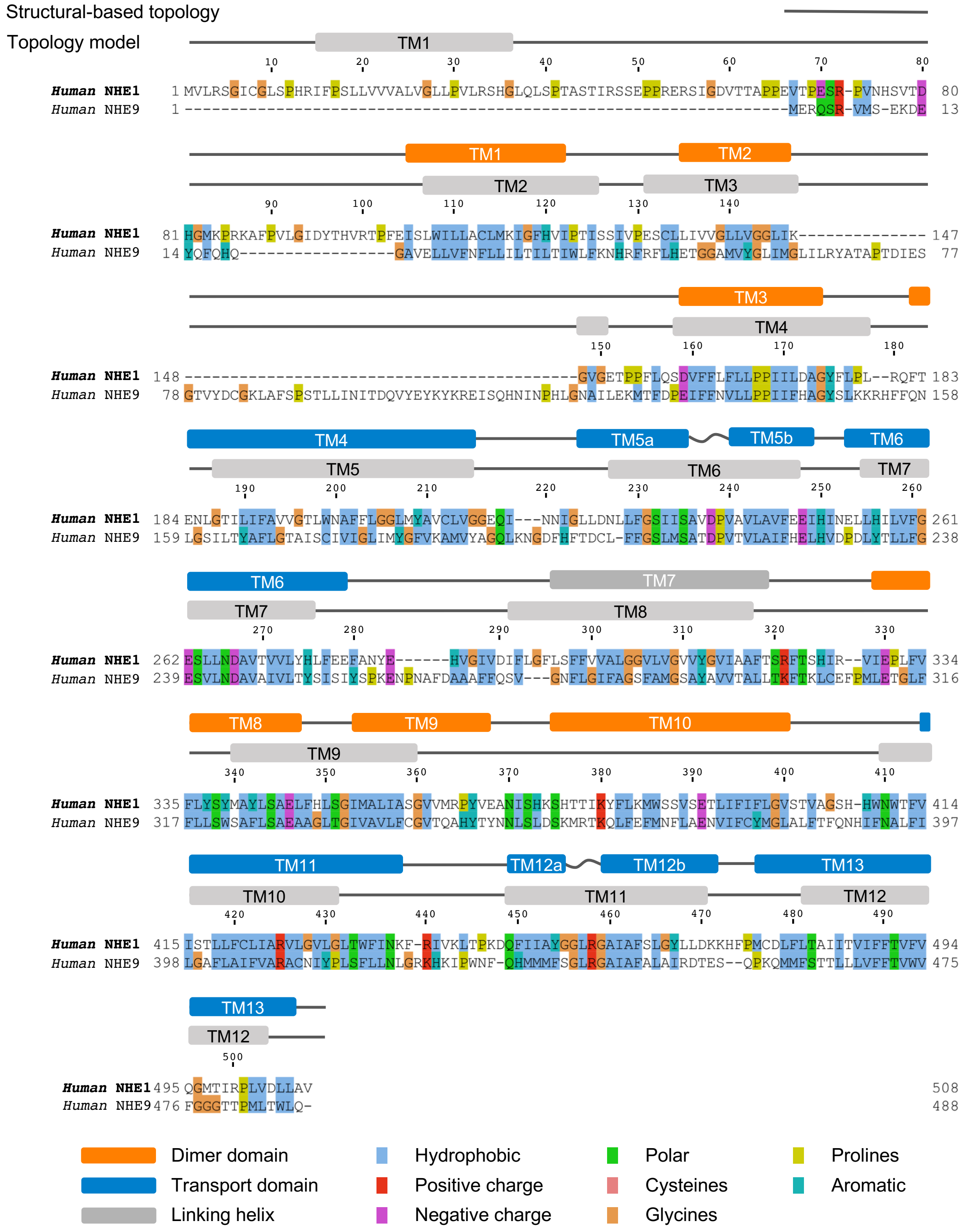

Appendix Figure S5. Structural-based alignment of NHE9 *vs.* NHE1 model. The most consensus topology for NHE1 (Physiol Rev 99, 2015-2113, 2019), includes a non-cleavable signal peptide and excludes the dimerization domain helices TM9 and TM10. NHE1 therefore most likely ha 13 TMs and as the first non-conserved hydrophobic stretch probably a cleavable signal peptide.

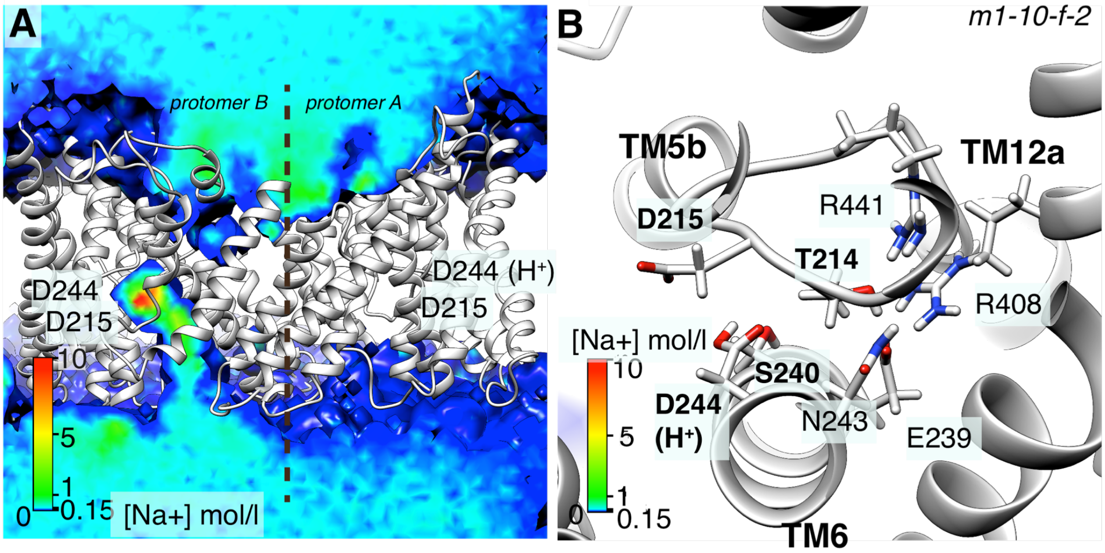

Appendix Figure S6. MD simulation of NHE9 with protonated and deprotonated binding site residue D244. (A) Na^+ density from MD simulation (m1-10-f-2), measured in mol/l. The bulk density is ~150 mM (cyan). D244 in *protomer A* (right) was simulated with a proton bound (i.e., neutral, state S1 in Supplementary Table 3), and no density above the cut-off of 0.001 mol/l (dark blue) was detected, indicative of no binding (as observed in all simulations with protonated D244). D244 in *protomer B* (left) was simulated in its charged (deprotonated) form (state S0 in Supplementary Table 3), and Na^+ entered the site spontaneously and approached D215 but in this simulation did not bind to D244 (even though complete spontaneous binding events were observed in other simulations). The membrane is omitted for clarity. **(B)** Top-view of the binding site with protonated D244 of the same; no density was detected near D244 and not even near the charged D215.

A

NHE9 intrinsic dynamics

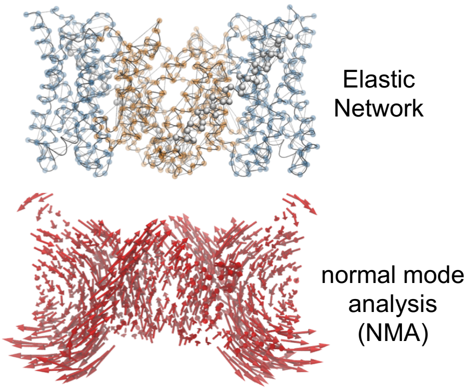

	NapA transition	NHE Family PC1	NapA inward NMA	NapA outward NMA	NHE9 inward NMA
**NapA transition**	100%	70%	92%	91%	82%
**NHE Family PC1**	70%	100%	70%	74%	70%
**NapA inward NMA**	92%	70%	100%	84%	75%
**NapA outward NMA**	91%	74%	84%	100%	70%
**NHE9 inward NMA**	82%	70%	75%	70%	100%

B

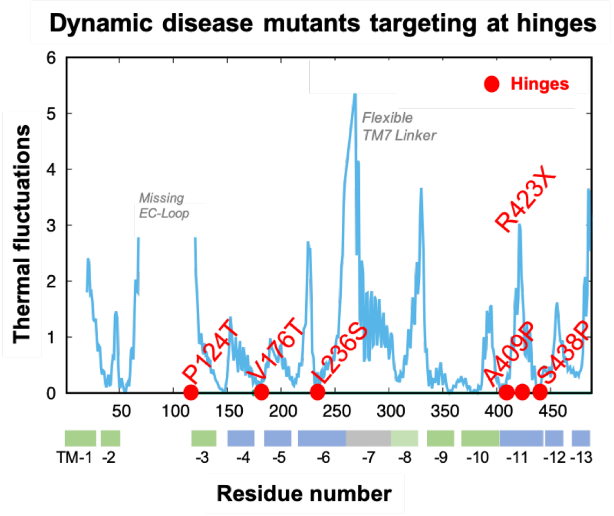

C

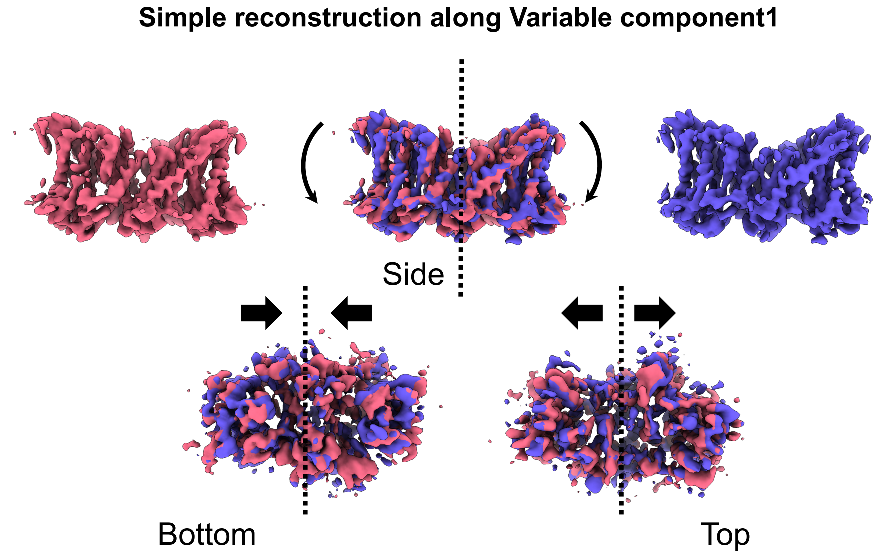

Appendix Figure S7. NHE9 dynamics as assessed by PCA, ENM and 3DVA (A) Comparison of Principle Component Analysis (PCA) and Elastic Modelling (ENM). *Left*: ENM for NHE9 (*top*) and front view of elevator-like NMs (*bottom*); *Right:* overlaps between experimental NapA inward-outward transition and NHE Family PC1 versus the first 20 Normal Modes computed from NHE9/NapA core structures (details in Materials and Methods). **(B)** Intrinsic dynamics of NHE9 and location of known disease mutants. Reported disease mutations are located in the hinges that facilitate the elevator transitions interconverting inward and outward states. Near-equilibrium residue fluctuations for NHE9 inward structure computed from the first 20 ENM normal modes describing the transition (Materials and Methods). Note how disease-associated mutations are selectively located at flexibility minima, which act as hinge regions for large rigid-body rearrangements. **(C).** 3D Variability analysis performed by cryoSPARC v2.14.2. Two opposite conformations are shown (dark red and dark blue) and where reconstructed along 'Variable component 1'. The movement between these conformations is depicted by black arrows. (top) "elevator like" conformational changes in the core domains. (bottom) "breathing" of the dimer domain.

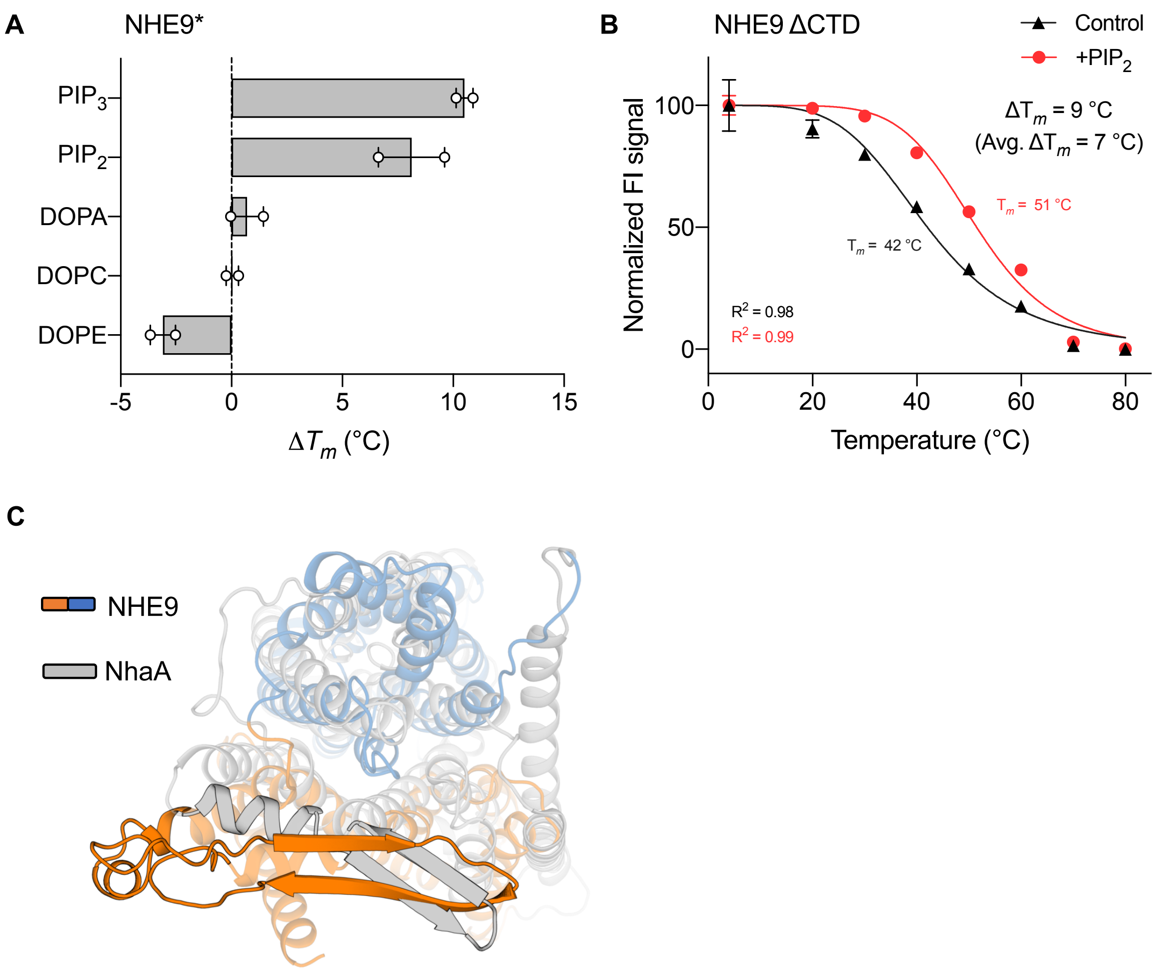

Appendix Figure S8. Lipid-interaction analysis of NHE9 and comparison of modelled TM2-TM3 loop to NhaA. (A) Average ΔT_m shifts for NHE9* calculated from thermal shift stabilization of purified dimeric NHE9*-GFP in the presence and absence of listed lipids, data are mean ± data range of n = 2 independent experiments. **(B)** Thermal shift stabilization of purified dimeric NHE9-ΔCTD-GFP in the presence of PIP_2 (red) compared to PIP_2-free (black). Data presented are normalized mean FSEC peak fluorescence as mean values ± data range of n = 2 technical repeats; the apparent T_m was calculated with a sigmoidal 4-parameter logistic regression function; the average ΔT_m presented is calculated from n = 2 independent titrations. **(C)** Extracellular view of a superimposition between the *E. coli* NhaA monomer (grey) and the NHE9 monomer loop model constituted by the core transport domain (blue) and the dimerization domain (orange). Notably, the TM2-TM3 loop is predicted to form a similar β-hairpin structure as seen in NhaA.

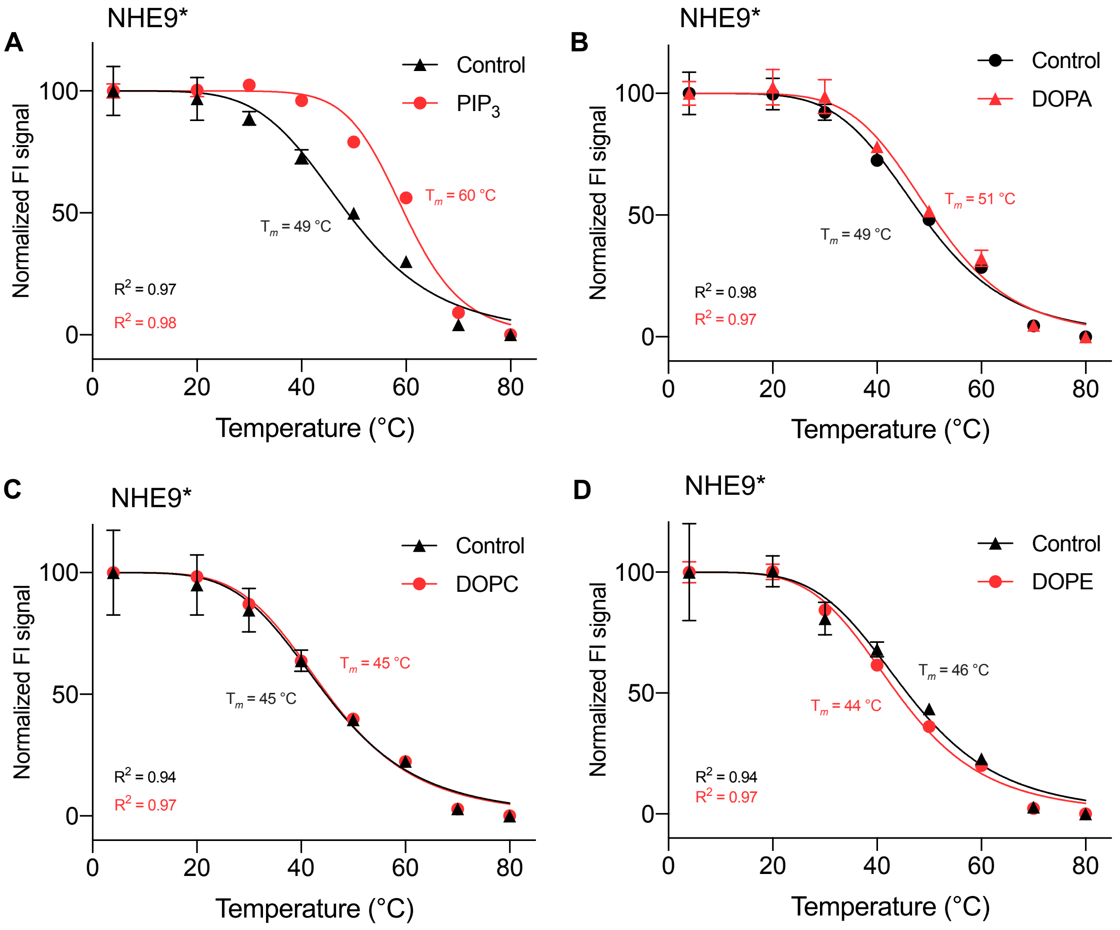

Appendix Figure S9. Thermal-shift melting curves for NHE9* after the addition of various lipids. (A-D) Normalized mean FSEC peak fluorescence of purified dimeric NHE9*-GFP over a range of eight temperature points in the absence (black) and presence (red) of (a) PIP_3, (b) DOPA, (c) DOPC, and (d) DOPE; data are mean ± data range of n = 2 technical repeats; the apparent T_m was determined with a sigmoidal 4-parameter logistic regression function.

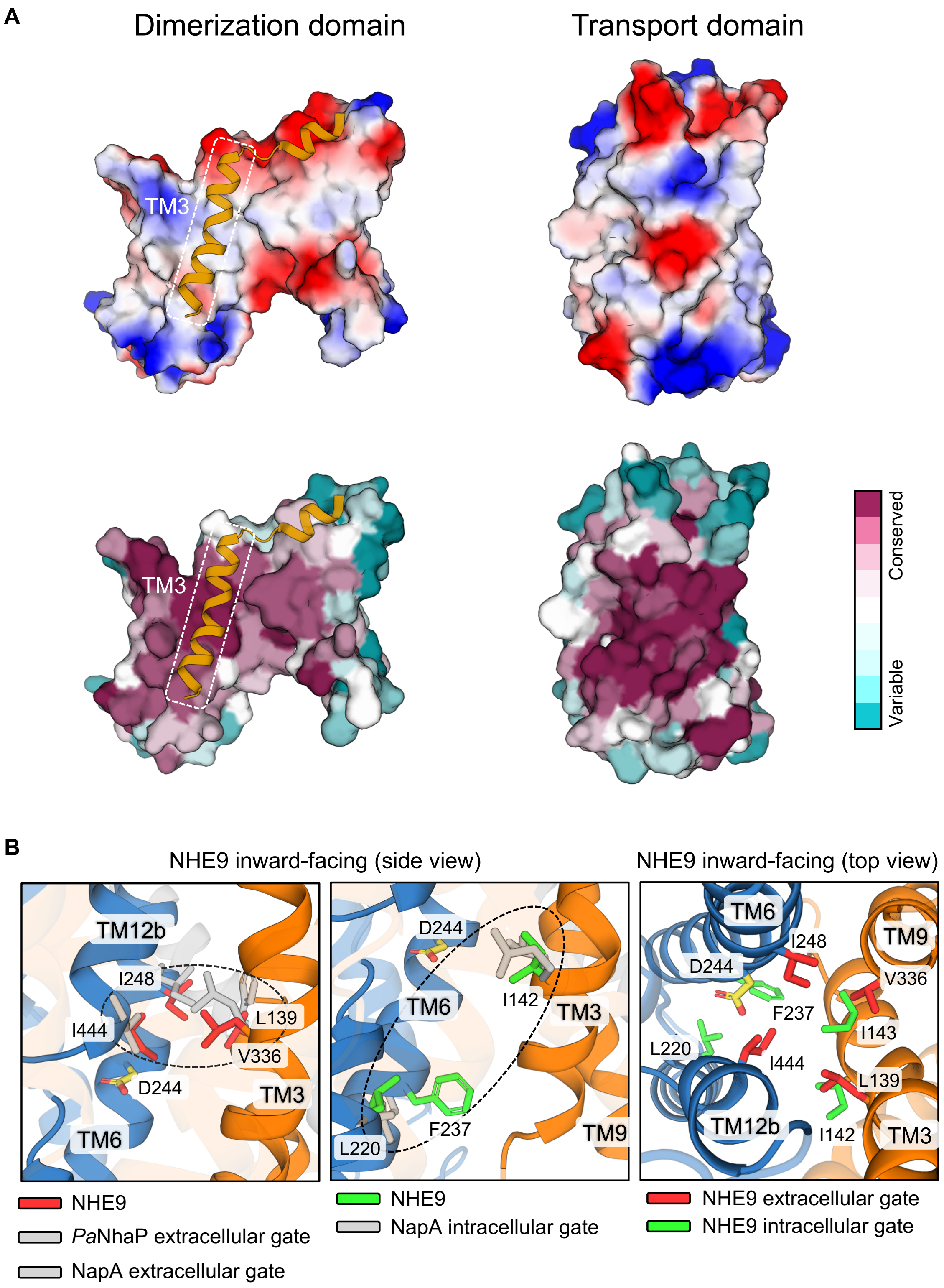
A
Dimerization domain
Transport domain
TM3
TM3
Conserved
Variable
B
NHE9 inward-facing (side view)
NHE9 inward-facing (top view)
TM12b
I248
I444
L139
V336
D244
TM3
TM6
D244
TM6
I142
TM3
F237
L220
TM9
TM6
I248
TM9
D244
V336
F237
I143
L220
I444
L139
TM12b
I142
TM3
NHE9
PaNhaP extracellular gate
NapA extracellular gate
NHE9
NapA intracellular gate
NHE9 extracellular gate
NHE9 intracellular gate

Appendix Figure S10. Hydrophobic interactions between the 6-TM core transport domains and the dimerization domain in NHE9. (A) Surface representation of the NHE9 dimer domain looking onto the interface between the dimerization domain (left) and core transport domain (right) as rendered by electrostatic properties (top) and ConSurf score (bottom) based on a sequence alignment of 155 sequences from the UniRef90 database (covering all kingdoms) (see Materials and Methods). TM3 of the dimer domain is shown in cartoon representation. Notably, (i) a highly conserved hydrophobic stretch, referred to as a hydrophobic barrier, is located on TM3 that faces the core transport domain interface and (ii) residues surrounding the negatively-charged ion-binding site in the core transport domain are also highly-conserved hydrophobic residues, which form extracellular and intracellular hydrophobic gates either side of the ion-binding site **(B)** *left:* view from the side showing the hydrophobic extracellular-gate formed between Ile248, Ileu444, Val336 and Leu139 for inward-facing NHE9 (red sticks) that is located above the ion-binding Asp244 (yellow stick form). The core transport domain is coloured blue and the dimerization domain is coloured orange. The extracellular hydrophobic gate is a conserved feature of all Na^+/H^+ antiporters (dotted ellipse) as shown here for the corresponding residues in the inward-facing NapA and *Pa*NhaP structures (grey sticks). *middle:* Ile142, Phe237 and Leu220 are modelled to come together to form an hydrophobic intracellular-gate (green sticks) upon transition to the outward-facing state (dotted ellipse) as based on the outward-facing state of NapA (grey sticks) and as modelled (Materials and Methods and Supplementary Video 3). Note, based on the sequence alignment from (A), all hydrophobic residues have conservation score of at least 8 (9 is the highest): Leu139 (9), Ile142 (9), Ile143 (9), Leu220 (9), Phe237 (9), Ile248 (9), Val336 (8), Ile444 (8).

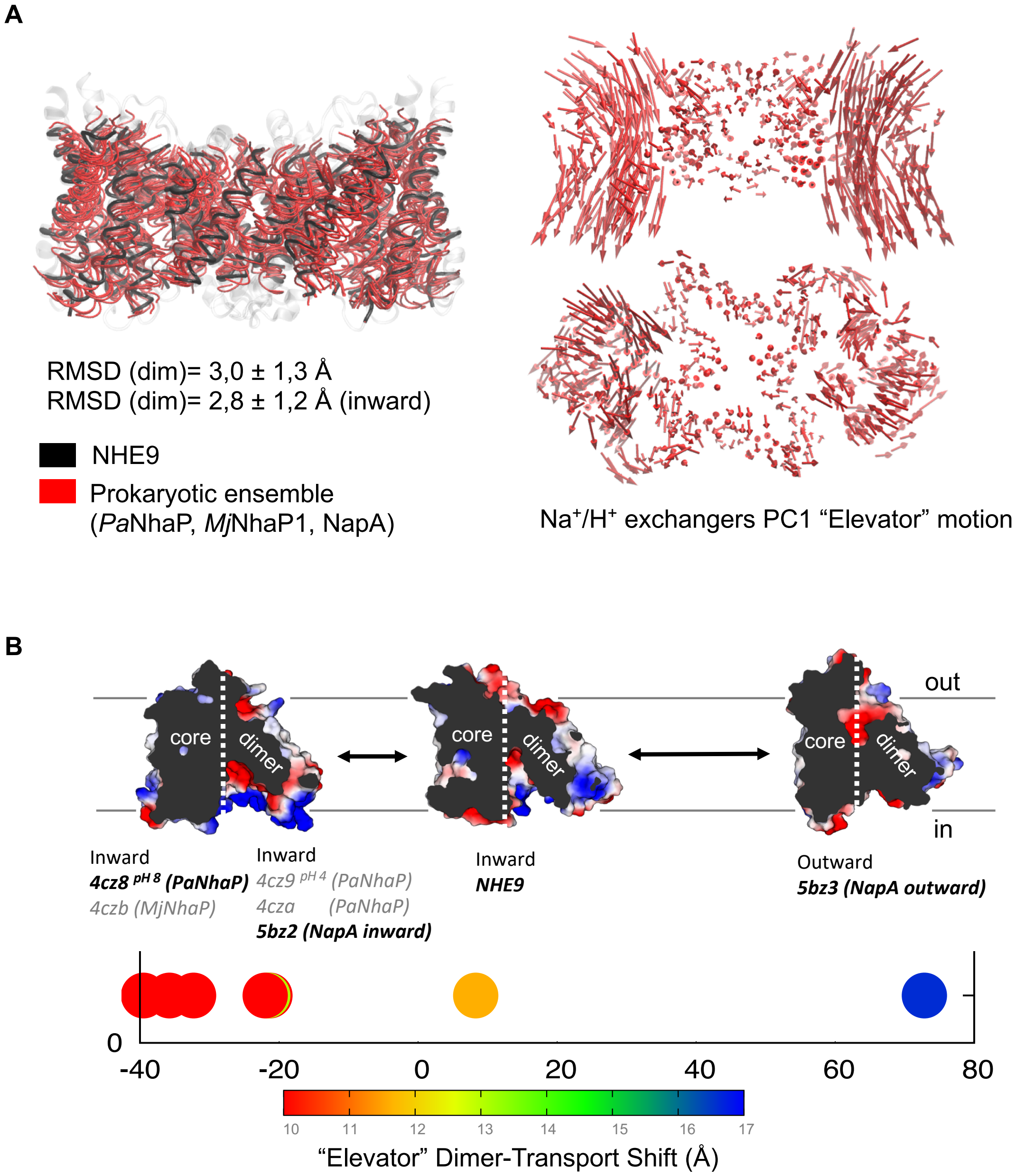
A
RMSD (dim)= 3,0 ± 1,3 Å
RMSD (dim)= 2,8 ± 1,2 Å (inward)
NHE9
Prokaryotic ensemble
(PaNhaP, MjNhaP1, NapA)
Na+/H+ exchangers PC1 “Elevator” motion
B
core
dimer
core
dimer
core
dimer
out
in
Inward
4cz8 pH 8 (PaNhaP)
4czb (MjNhaP)
Inward
4cz9 pH 4 (PaNhaP)
4cza (PaNhaP)
5bz2 (NapA inward)
Inward
NHE9
Outward
5bz3 (NapA outward)
0
-40
-20
0
20
40
60
80
10
11
12
13
14
15
16
17
“Elevator” Dimer-Transport Shift (Å)

Appendix Figure S11. (A) Principal component analysis from the ensemble of Na^+/H^+ exchangers with known structures. *Right:* Structurally conserved core for NHE9, NapA, *Pa*NhaP and *Mj*NhaP1 structures (n = 8 dimers from 7 PDB codes). *Left:* Front (top) and top (bottom) view of the major mode from PCA (PC1), which captures the majority of the elevator-transition between inward and outward states (55% of the total structural variance of the ensemble). **(B)** Projections of NHE Family structures onto the major component (PC1, $Å^2$), coloured according to their elevator-like structural shift. Note how PC1 efficiently distinguishes outward from inward states, the latter being further clustered into distinct inward states. At the top, membrane transversal cross sections of surface representation of different NHE monomers viewed through the ion-translocation funnel, showing the intermediate shift for NHE9. From left to right: *Pa*NhaP, pH 8 (PDB id:4cz8), *Mj*NhaP (PDB id: 4czb), NapA inward (PDB id: 5bz2), NHE9 inward, NapA outward (PDB id: 5bz3). PC1 color scale reports the elevator-shift estimated as the distance between Asp244 and the gate residue Ile142; the pure Cα vertical displacement of the ion-binding Asp244 between inward and outward NapA states is 8 Å.

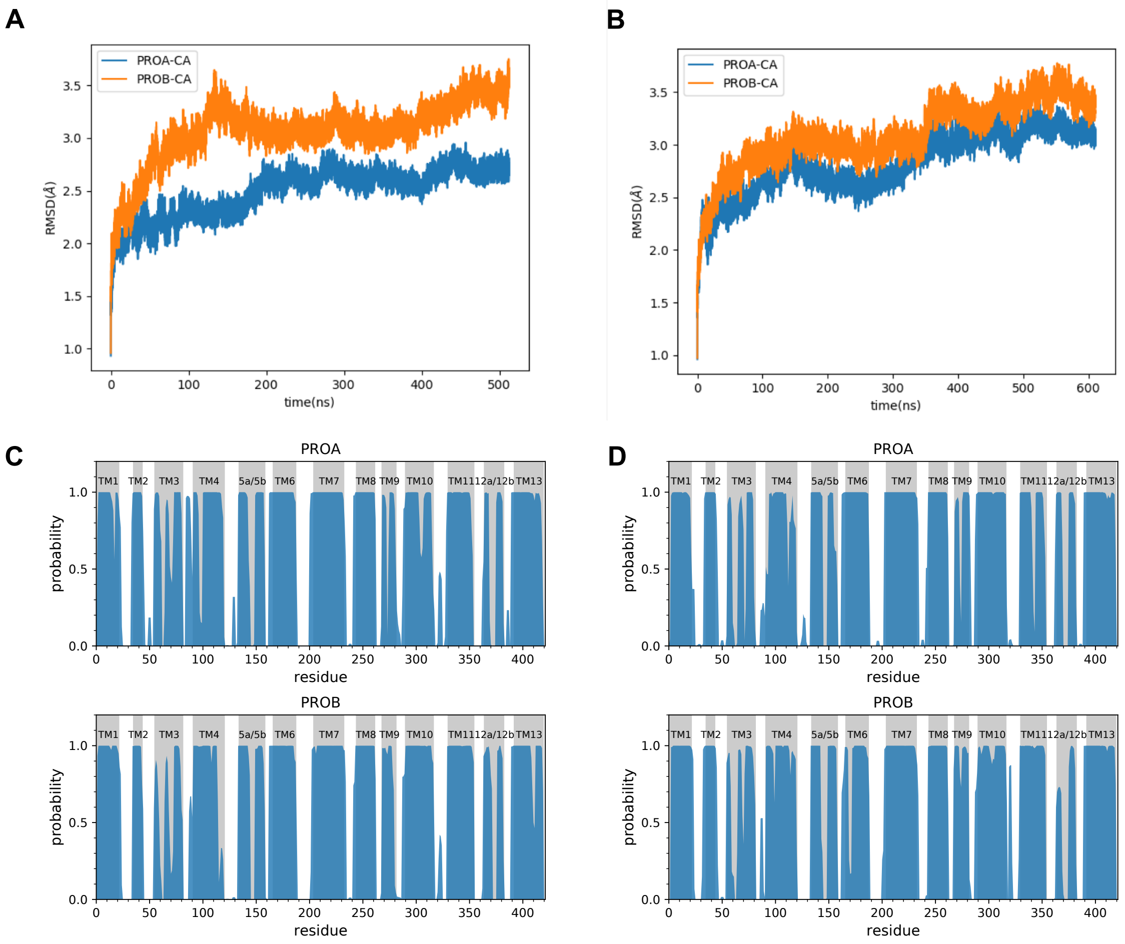

Appendix Figure S12. Structural analysis of selected MD simulations. (A) Root mean square displacement (RMSD) of transmembrane helices after optimal superposition of the Cα atoms relative to the experimental cryo-EM structure, calculated separately for protomer A (PROA) and protomer B (PROB); data for simulation m2-00-p-2 (see Supplementary Table 3). **(B)** RMSD for simulation m2-00-f-3. **(C)** Secondary structure per residue (DSSP) probability (blue), calculated as fraction of the total simulation time (simulation m2-00-p-2) and shown for each protomer separately. Helices as observed in the cryo-EM structure are indicated by gray boxes. Residues are numbered continuously as for the simulated model (see text). **(D)** Secondary structure probability (as in c.) for simulation m2-00-f-3.
