# Supplementary material for: Structure and elevator mechanism of the mammalian sodium/proton exchanger NHE9
Source: EMBO J. 2020 Oct 29;39(24):e105908. doi: 10.15252/embj.2020105908 (PMC7737618; doi:10.15252/embj.2020105908)
Supplement: Supplementary file 2 — Expanded View Figures PDF [file EMBJ-39-e105908-s002.pdf]

Expanded View Figures

Figure EV1. Multiple sequence alignment of *horse* NHE9 and *human* NHE1-9 isoforms.
Residues with over 70% sequence identity are indicated by purple background. Conserved ion-binding site residues are highlighted with a red border. Positions which have been identified to harbour disease mutations in *human* NHE9 are indicated with an asterisk (*). Residues which are predicted by SignalP-5.0 (likelihood 0.56–0.98) to be part of a single peptide at the N-terminus are encircled with a dashed red box. Helix breakpoints (s-shaped line), connecting loops (lines), non-modelled loops (dashed line), dimer domain TMs (orange), core 6-TM transport domain TMs (blue) and the linking helix TM7 (grey) are indicated.

© 2020 The Authors

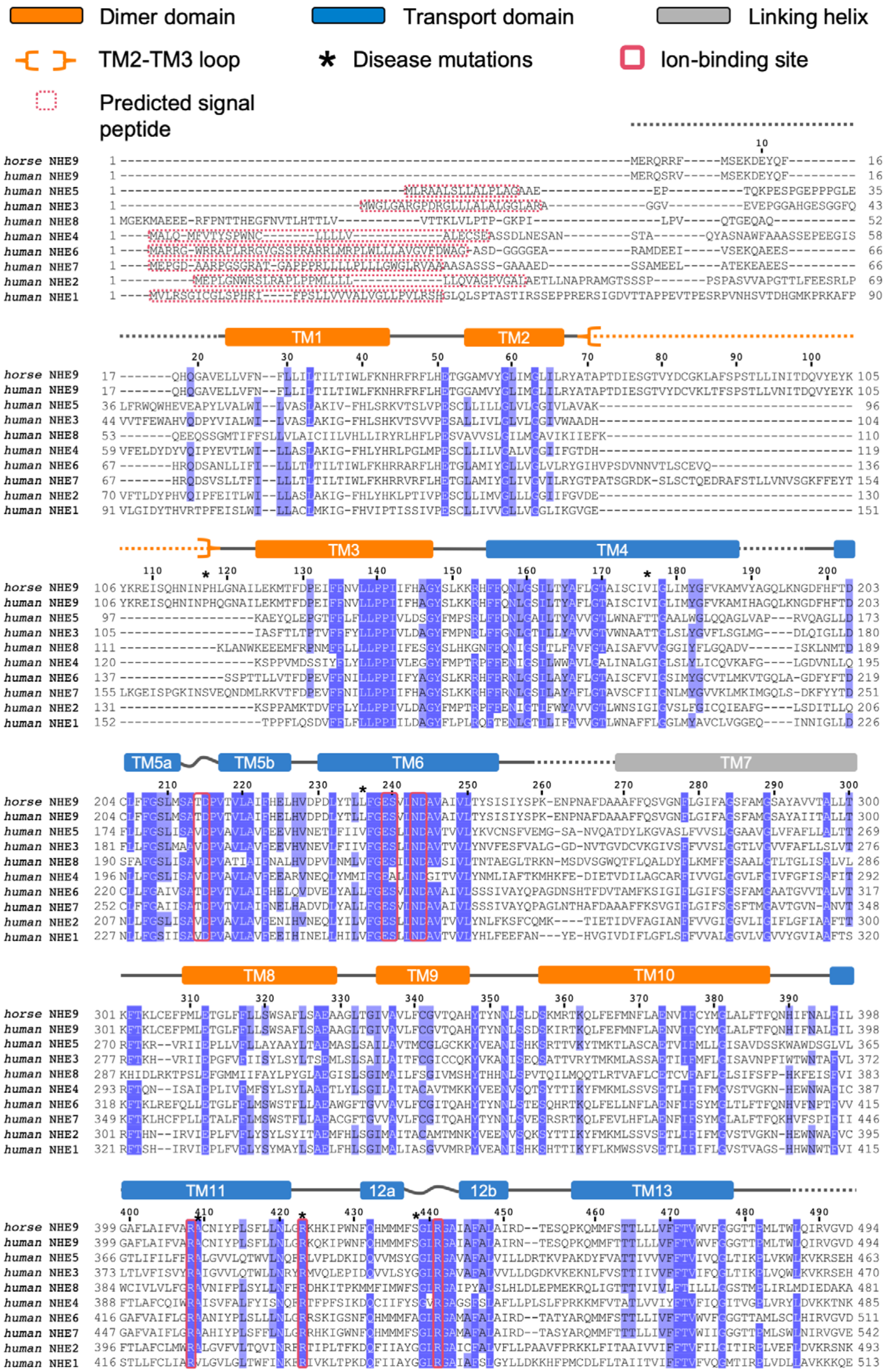
Dimer domain
Transport domain
Linking helix
TM2-TM3 loop
Disease mutations
Ion-binding site
Predicted signal peptide
horse NHE9
human NHE9
human NHE5
human NHE3
human NHE8
human NHE4
human NHE6
human NHE7
human NHE2
human NHE1
TM1
TM2
TM3
TM4
TM5a
TM5b
TM6
TM7
TM8
TM9
TM10
TM11
12a
12b
TM13

Figure EV1.

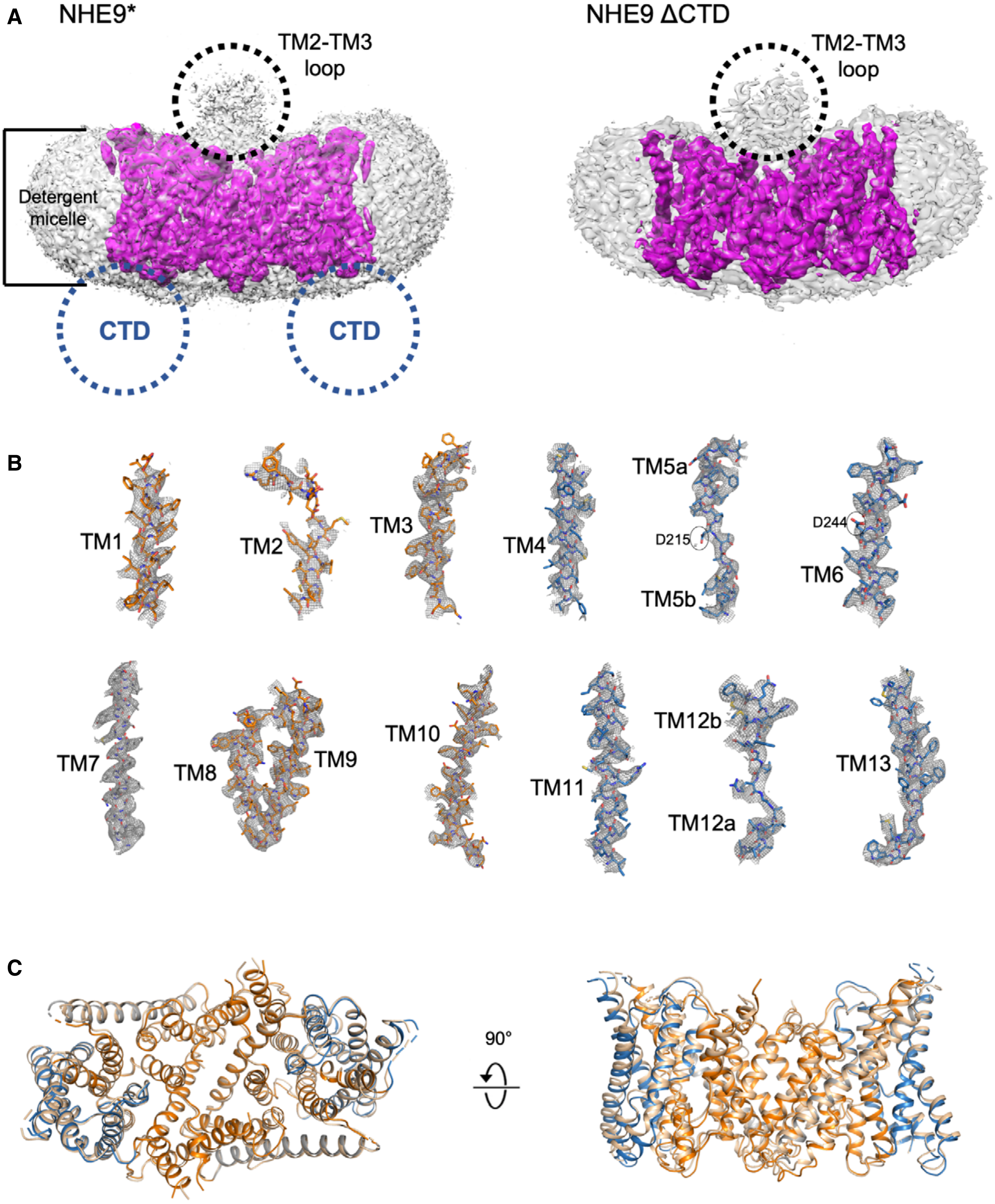

Figure EV2. Cryo-EM density of horse NHE9* and NHE9 ΔCTD before and after density subtraction.

A NHE9* (left purple) and NHE9 ΔCTD (right purple) structures docked into their respective cryo-EM density maps before micelle density subtraction. Non-modelled cryo-EM density for the 51-residue long TM2–TM3 loop was apparent at the dimer interface (black-dotted circle). The NHE9* construct contains 93 out of 163 residues of the C-terminal regulatory domain (blue-dotted circle), but no corresponding additional density was apparent as compared to the NHE9 ΔCTD density maps.

B Cryo-EM density map and model are shown for all transmembrane segments for NHE9 ΔCTD in the dimer domain (orange), transport domain (blue) and linker TM7 (grey). Residues D215 and D244 (encircled) on TM5 and TM6, respectively, have been modelled after *Pa*NhaP at pH 8.0 (PDB id: 4cz8).

C Superimposition of NHE9* (wheat) and NHE9 ΔCTD (core transport domains (blue), dimerization domains (orange), linker helices (grey)) structures, which show only small differences in the most mobile regions.

 © 2020 The Authors

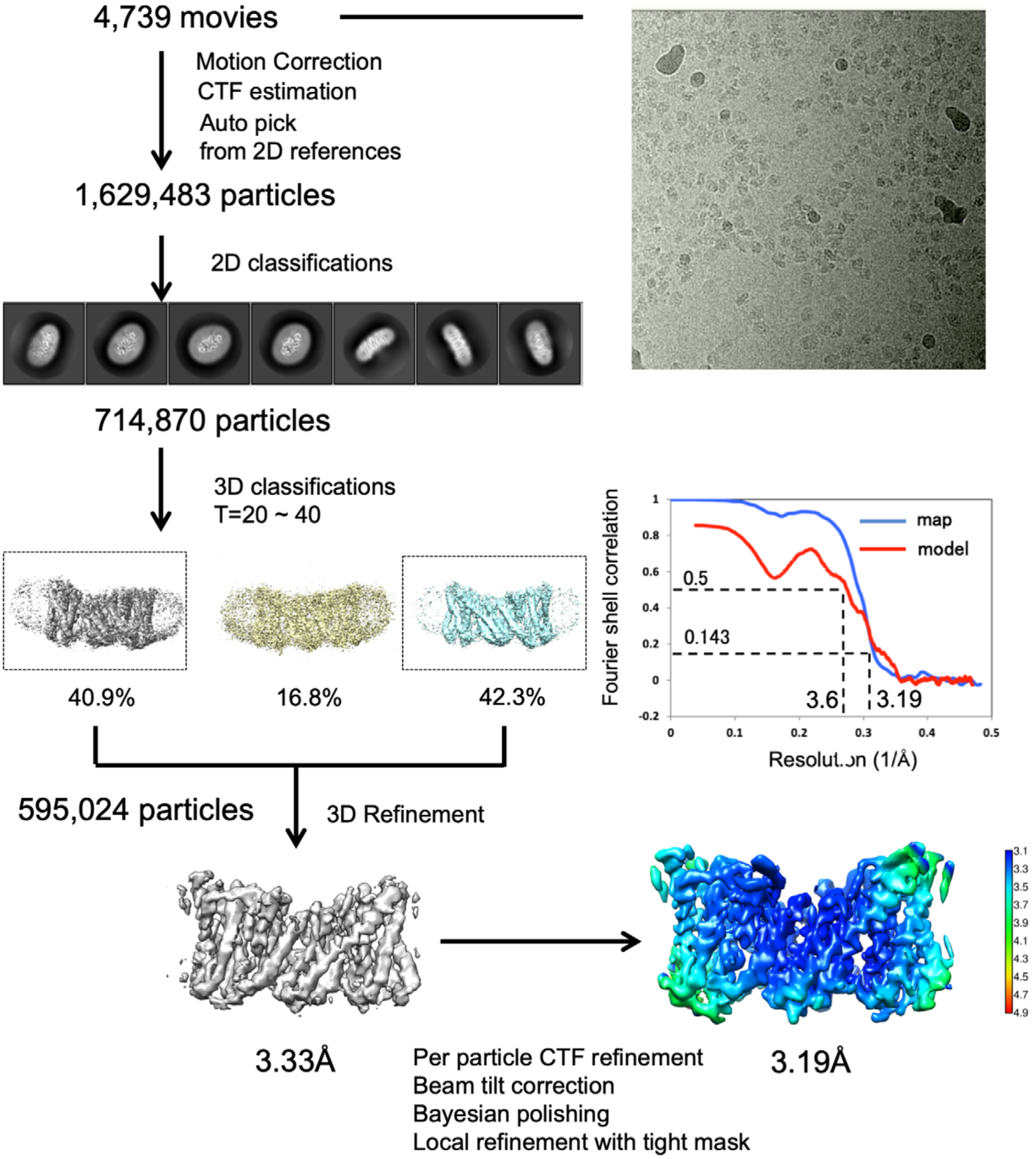

Figure EV3. The data-processing workflow of *horse* NHE9 ΔCTD.
The dataset contained 4,739 movies that were corrected by MotionCor2 and CTFFind. After reference-based auto-picking, 1,629,483 particles were picked. Several rounds of 2D classification were performed, yielding 714,870 particles, which were subjected to 3D classification. One of the three 3D classes was selected, and it contained 595,024 particles. After several rounds of refinement with global and local search using ctf refine and polishing, a final resolution of 3.19 Å was achieved at gold-standard FSC (0.143), with a local resolution range of 3.1–4.1 Å.

Figure EV4. Cross-sectional comparison of NHE9 against Na^+/H^+ antiporters and CitS.

A Slice through electrostatic surface representation of *horse* NHE9 and bacterial homologues as labelled. The more distantly related inward-facing structure of the citrate transporter CitS is also shown as a further example of cavity depth and hydrophobic gap located between protomers. The approximate buried surface area between monomers shown was calculated with PBDePISA.

B Electrostatic surface representation of the cytoplasmic view of the inward-facing NHE9 monomer (left) and the NHE1 model based on the NHE9 structure (right).

© 2020 The Authors

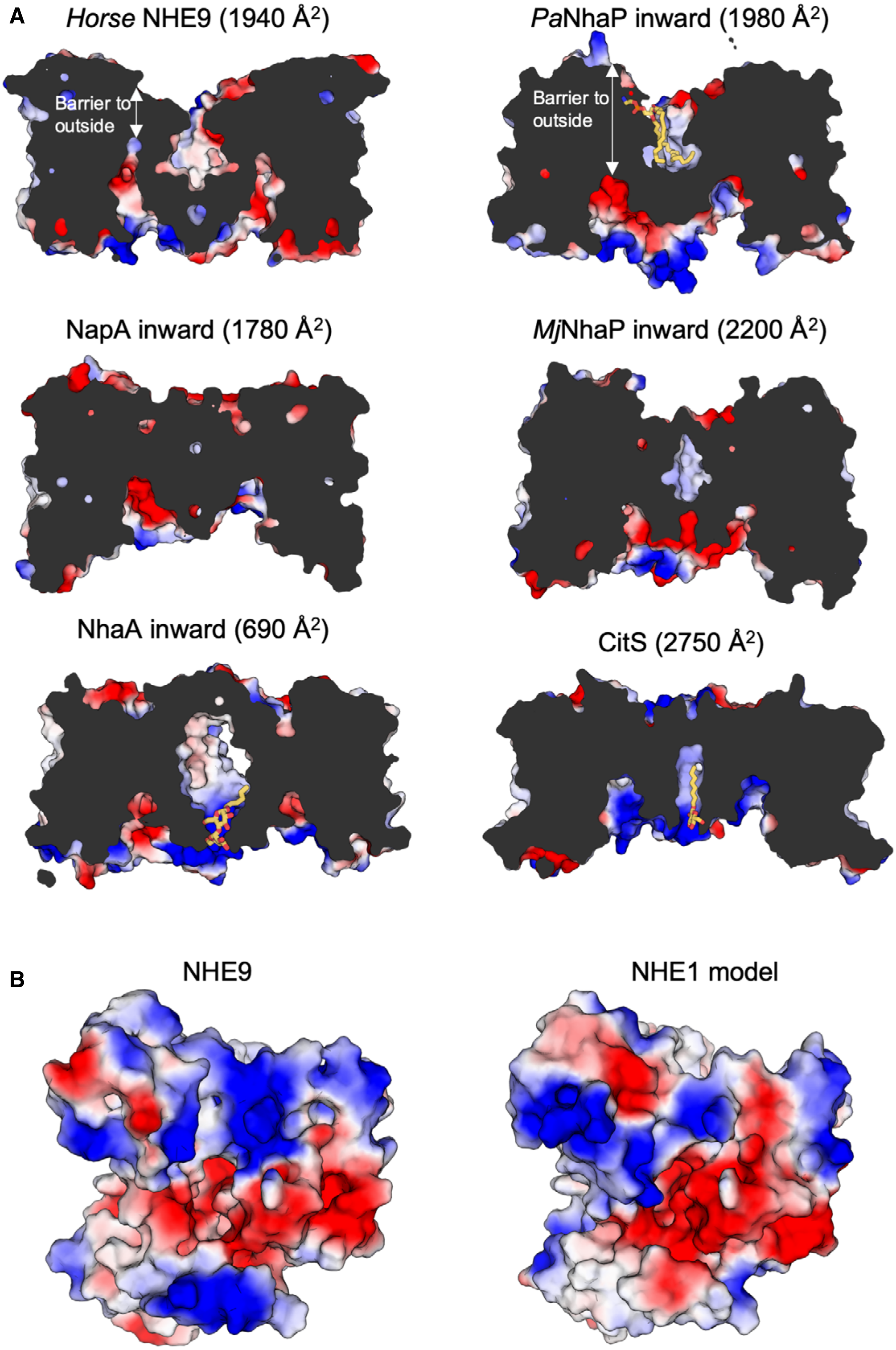
A
Horse NHE9 (1940 Å2)
PaNhaP inward (1980 Å2)
Barrier to outside
Barrier to outside
NapA inward (1780 Å2)
MjNhaP inward (2200 Å2)
NhaA inward (690 Å2)
CitS (2750 Å2)
B
NHE9
NHE1 model

Figure EV4.

Figure EV5. NHE9 ion-binding site and location of disease mutations.

A Cartoon representation of the NHE9 6-TM core transport domain. The crossover of broken helices TM5a and TM5b (green) and TM12a and TM12b (purple) is unique to the NhaA-fold and the half-helical dipoles that they create are highlighted. Between the half-helical dipoles are oppositely charged and strictly conserved residues located in the peptide break region of all NHE isoforms (see ED Fig 1) shown here in stick form.

B Comparison between the ion-binding site of NHE9 (yellow, numbered) and *Pa*NhaP (light grey, PDB id: 4cz9) is indicated. The Tl^{+} ion in *Pa*NhaP (purple sphere) is further coordinated by the glutamic acid E73 residue (grey sticks; labelled with *) not conserved in NHEs. Further glutamic acid residue D93 in *Pa*NhaP and *Mj*NhaP (light grey and dark grey sticks, respectively; labelled with *) in TM4 is also not conserved in the NHEs, but extends towards the conserved Asn243 in NHE9 reflecting the ion-binding site differences between bacterial electroneutral Na^{+}/H^{+} antiporters and mammalian NHEs.

C In electroneutral Na^{+}/H^{+} antiporters, a salt bridge is formed between residues corresponding to Glu239 and Arg408 in NHE9 (yellow sticks). In the electrogenic Na^{+}/H^{+} antiporters, a salt bridge is instead formed between an aspartic acid (Asn243 in NHE9) and a lysine residue (Arg408 in NHE9) as shown here for NapA (grey sticks).

D Cartoon representation of the NHE9 monomer with the 6-TM core transport domain (blue), dimerization domain (orange) and linker helix TM7 (grey) shown from the side (left) and top (right). Conserved proline and glycine residues are shown as yellow spheres. Residues which have been identified to harbour autism disease mutations in patients are shown as red spheres and labelled.

© 2020 The Authors

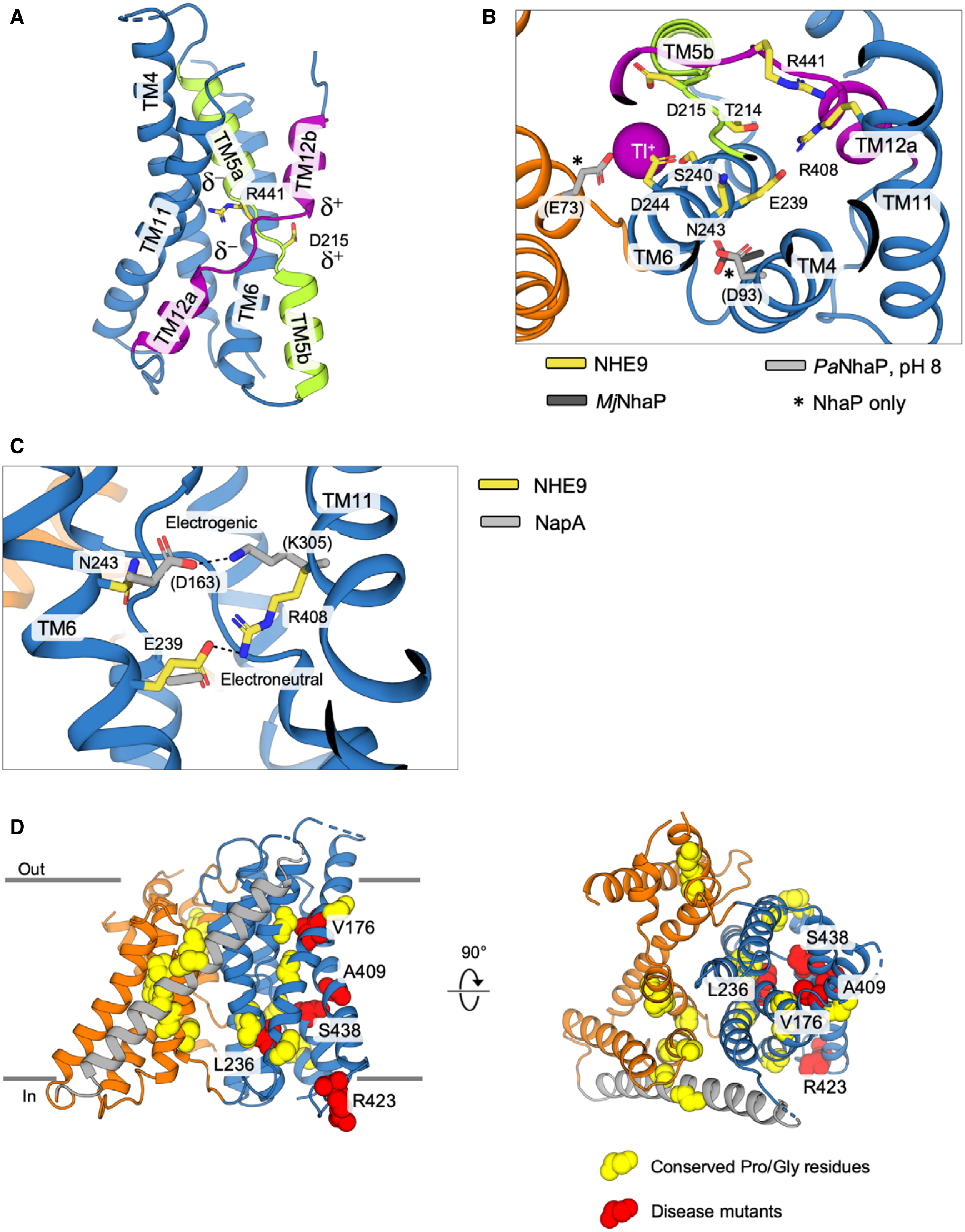
A
TM4
TM5a
TM12b
δ−
R441
δ+
TM11
δ−
D215
δ+
TM12a
TM6
TM5b
B
TM5b
R441
D215
T214
Tl+
TM12a
R408
S240
(E73)
D244
E239
TM11
N243
TM6
TM4
(D93)
NHE9
PaNhaP, pH 8
MjNhaP
* NhaP only
C
TM11
Electrogenic
(K305)
N243
(D163)
R408
TM6
E239
Electroneutral
NHE9
NapA
D
Out
V176
A409
S438
L236
In
R423
90°
S438
L236
A409
V176
R423
Conserved Pro/Gly residues
Disease mutants

Figure EV5.
