# Supplementary material for: Structure and elevator mechanism of the mammalian sodium/proton exchanger NHE9
Source: EMBO J. 2020 Oct 29;39(24):e105908. doi: 10.15252/embj.2020105908 (PMC7737618; doi:10.15252/embj.2020105908)
Supplement: Supplementary file 3 — Movie EV1 [file EMBJ-39-e105908-s003.zip › Movie_EV1_legend.docx]

**Movie EV1**

Movie depicting the distribution of discrete states in the final 3D reconstruction of the *horse* NHE9 ΔCTD structure obtained by cryo-EM using the algorithm 3D Variability Analysis (Methods).
