# Supplementary material for: Structure and elevator mechanism of the mammalian sodium/proton exchanger NHE9
Source: EMBO J. 2020 Oct 29;39(24):e105908. doi: 10.15252/embj.2020105908 (PMC7737618; doi:10.15252/embj.2020105908)
Supplement: Supplementary file 4 — Movie EV2 [file EMBJ-39-e105908-s004.zip › Movie_EV2_legend.docx]

**Movie EV2**

Movie depicting the NHE9 intrinsic dynamics calculated by normal mode analysis from the inward-facing *horse* NHE9 ΔCTD structure. Normal Modes from NHE9 display elevator-like motions that displace the ion binding Asp244 as the hydrophobic dimer and transport interface gates rearrange.
