# Supplementary material for: Structure and elevator mechanism of the mammalian sodium/proton exchanger NHE9
Source: EMBO J. 2020 Oct 29;39(24):e105908. doi: 10.15252/embj.2020105908 (PMC7737618; doi:10.15252/embj.2020105908)
Supplement: Supplementary file 5 — Movie EV3 [file EMBJ-39-e105908-s005.zip › Movie_EV3_legend.docx]

**Movie EV3**

Major Principal Component analysis of the sodium/proton exchanger ensemble, showing the conserved ion-binding aspartate (red) and the hydrophobic gates at the interface between the dimer (purple) and transport (yellow) domains. Note how the hydrophobic gates rearrange to enable access to the ion-binding aspartate to each side of the membrane.
